# Distributed Sparse Manifold-Constrained Optimization Algorithm in Linear Discriminant Analysis

**DOI:** 10.3390/jimaging11030081

**Published:** 2025-03-13

**Authors:** Yuhao Zhang, Xiaoxiang Chen, Manlong Feng, Jingjing Liu

**Affiliations:** 1 State Key Laboratory of Integrated Chips and Systems, School of Microelectronics, Fudan University, Shanghai 200433, China; 21112020123@m.fudan.edu.cn (Y.Z.); 21112020077@m.fudan.edu.cn (X.C.); 2 Shanghai Key Laboratory of Automobile Intelligent Network Interaction Chip and System, School of Microelectronics, Shanghai University, Shanghai 200444, China

**Keywords:** distributed sparse manifold constraint (DSC), linear discriminant analysis (LDA), manifold proximal gradient (ManPG), non-convex sparse optimization

## Abstract

In the field of video image processing, high definition is one of the main directions for future development. Faced with the curse of dimensionality caused by the increasingly large amount of ultra-high-definition video data, effective dimensionality reduction techniques have become increasingly important. Linear discriminant analysis (LDA) is a supervised learning dimensionality reduction technique that has been widely used in data preprocessing for dimensionality reduction and video image processing tasks. However, traditional LDA methods are not suitable for the dimensionality reduction and processing of small high-dimensional samples. In order to improve the accuracy and robustness of linear discriminant analysis, this paper proposes a new distributed sparse manifold constraint (DSC) optimization LDA method, called DSCLDA, which introduces $L_{2,0}$-norm regularization for local sparse feature representation and manifold regularization for global feature constraints. By iterating the hard threshold operator and transforming the original problem into an approximate non-convex sparse optimization problem, the manifold proximal gradient (ManPG) method is used as a distributed iterative solution. Each step of the algorithm has an explicit solution. Simulation experiments have verified the correctness and effectiveness of this method. Compared with several advanced sparse linear discriminant analysis methods, this method effectively improves the average classification accuracy by at least 0.90%.

## 1. Introduction

An ultra-high-definition video image processing system relies on the ability to detect information from multiple targets and over long distances [1,2]. However, the current chip computing power cannot support complex computational imaging processing methods, resulting in the inability to meet the real-time requirements of the system, and the processing performance has not yet reached the demand for high-dimensional information detection and perception. Therefore, it is necessary to quickly and adaptively process high-dimensional video images. Nowadays, complex image data are inherently redundant and non-Gaussian, leading to the unstable performance of traditional methods such as principal component analysis (PCA), linear discriminant analysis (LDA) [3,4], Fisher discriminant analysis (FDA) [5], orthogonal linear discriminant analysis (OLDA) [6], and uncorrelated linear discriminant analysis (ULDA) [7], which has affected the actual video processing performance. Therefore, it is urgent to explore how to utilize high-dimensional spatial data, establish new sparse discrimination models, and design effective optimization schemes to improve existing detection and classification strategies.

From the perspective of data analysis, the key to processing and analyzing high-dimensional data lies in dimensionality reduction and feature extraction, with a focus on sparsity [8,9]. As an emerging optimization branch, sparse constraints have attracted much attention due to their ability to break through traditional Shannon sampling and achieve efficient transmission. Nowadays, sparsity constraints have been widely used in pattern recognition image processing, and its applicability in many other fields has been recognized [10,11]. Sparse constraints refer to the majority of elements being zero. For high-dimensional data, it is necessary to consider sparsity, such as through sparse linear discriminant analysis (SLDA) [12], sparse uncorrelated linear discriminant analysis (SULDA) [13], robust sparse linear discriminant analysis (RSLDA) [14], intra-class and inter-class kernel constraints (IIKCs) [15], hypergraph Laplacian-based semi-supervised discriminant analysis (HSDAFS) [16], adaptive and fuzzy locality discriminant analysis (AFLDA) [17], etc. Compared with traditional LDA methods, sparse discriminant analysis greatly improves the identification ability of the system. However, sparse discriminant analysis methods usually replace the $L_{1}$-norm with the $L_{0}$-norm to obtain convex optimization problems. Obviously, the $L_{0}$-norm can select the most representative feature variables and optimize faster than $L_{1}$-norm-constrained optimization. Examples include a sparse signal recovery framework based on segmented threshold $L_{0}$ gradient approximation [18], image non-negative matrix factorization with alternating smooth $L_{0}$-norm constraints [19], and sparse feature selection based on fast embedding spectral analysis [20]. The above methods also prove that the $L_{0}$-norm does indeed have better feature vector selection and faster optimization algorithm capabilities.

From the perspective of feature extraction, sparse analysis-based methods have significant data analysis capabilities but can not reveal potential causal relationships between variables during the analysis process [21,22,23]. To address this problem, manifold learning can be introduced to learn local features of potential information in high-dimensional space. To characterize such data, a practicable solution is to map the linear inseparable features in high-dimensional space to a low-dimensional nonlinear feature space, such as through robust sparse manifold discriminative analysis (RSMDA) [24], which captures both global and local geometric information through manifold learning. Zhang et al. [25] proposed a coupled discriminative manifold alignment (CDMA) method, which focuses on aligning the manifold structures of a low resolution (LR) and high resolution (HR) in a common feature subspace. In order to use manifold learning methods, many optimization schemes have been proposed, such as the projection algorithm [26], precise penalty algorithm [27], augmented Lagrangian algorithm [28], iterative hard threshold [29], Newton hard threshold pursuit [30], etc. In addition, in the field of video image processing, the problem of high-dimensional pixel videos essentially requires a minimization optimization with Stiefel manifolds, including the partial least squares [31], principal component analysis [32], and canonical correlation analysis [33]. Manifold-constrained optimization can even be seen frequently in reinforcement learning [34] and federated learning [35].

The optimization methods mentioned above mostly focus on a single constraint. Currently, there is limited research on problems that consider both manifold constraints and sparse constraints. On the one hand, this is because both constraints are non-convex, non-smooth, and even NP-hard, making joint algorithm design difficult. On the other hand, joint constraints require two constraints to share the same variable, making theoretical analysis more difficult. To solve these difficulties, this paper proposes a new distributed sparse manifold-constrained optimization algorithm and explores effective numerical solutions. The proposed joint constraints are introduced to LDA, and the novel method is called distributed sparse manifold-constrained linear discriminant analysis (DSCLDA). The proposed method first divides the process monitoring data into multiple data nodes and performs distributed parallel operations simultaneously. Afterward, a $L_{2,0}$-norm sparse constraint is constructed to regulate local features and preserve the local structure of variables. In addition, by using manifold constraints on global variables, the proposed method can capture the causal correlation and reduce the data structure loss during the projection. By using the manifold proximal gradient (ManPG) to combine local and global variables, sparse constraints and manifold constraints are incorporated into the calculation process during optimization, and explicit solutions for each variable are obtained. The contributions of the proposed method can be presented as follows:

- This paper proposes a novel distributed sparse manifold-constrained linear discriminant analysis (DSCLDA) method, which introduces sparse and manifold constraints to maintain the local and global structure.
- We designed an effective solution scheme that combines local and global variables using the manifold proximal gradient (ManPG) to obtain explicit solutions for each subproblem.
- We conducted a series of experiments on several public datasets to verify the effectiveness of the proposed method and discuss the convergence and feature distribution.

The rest of this paper is organized as follows. Section 2 introduces the notations and related works. Section 3 details the problem of the proposed method and the corresponding optimization algorithm. Section 4 evaluates and discusses the performance of the proposed method. Section 5 concludes this paper.

## 2. Notations and Preliminaries

### 2.1. Notations

For convenience, we will define some symbols required for this section. For the matrix $X\in R^{n\times p}$, $X_{i}$ is represented as the *i*th row, and $X_{ij}$ is represented as an element of the *i*th row and *j*th column. $O_{n\times p}$ is written as an all-zero matrix in n×p, $I_{n\times p}$ represents the identity matrix with the dimensions n×p, and $I_{p}$ represents the identity matrix with the dimensions p×p. $X^{⊤}$ represents the transpose of *X*, and vec(X) represents the vectorization of *X*. For set T, $\bar{T}$ is used to represent the complement of *T*. In addition, for matrices $X,Y\in R^{n\times p}$, the inner product is defined as $\langle X,Y\rangle =\mathrm{tr}\left(X^{⊤}Y\right)=\sum_{i=1}^{n}\sum_{i=1}^{p}X_{ij}Y_{ij}$, where tr(·) represents the trace of the matrix.

### 2.2. Preliminaries

LDA, as a supervised learning method, can use prior experience of categories in the dimensionality reduction process, while unsupervised learning cannot use prior experience of categories. Compared with other methods, a feature of LDA is to learn discriminative projections by maximizing the inter-class distance while minimizing the intra-class distance, thereby achieving a more effective dimensionality reduction ability. Define the inter-class distance matrix $S_{b}$ and intra-class distance matrix $S_{w}$ for the training samples, and these two matrices can be defined as

(1) $$S_{b}=\frac{1}{n}\sum_{i=1}^{c}n_{i}\left({\bar{x}}_{i}-\bar{x}\right){\left({\bar{x}}_{i}-\bar{x}\right)}^{⊤},$$

(2) $$S_{w}=\frac{1}{n}\sum_{i=1}^{c}\sum_{j=1}^{n_{i}}\left({\bar{x}}_{ij}-{\bar{x}}_{i}\right){\left({\bar{x}}_{ij}-{\bar{x}}_{i}\right)}^{⊤}.$$

LDA attempts to find a suitable projection direction that minimizes intra-class dispersion and maximizes inter-class dispersion after projection. This search process can be expressed as follows:

(3) $$X=\underset{X^{⊤}X=I}{\arg\max}\frac{\mathrm{Tr}\left(X^{⊤}S_{b}X\right)}{\mathrm{Tr}\left(X^{⊤}S_{w}X\right)}.$$

To avoid the distortion of $S_{w}$, problem (3) can also be extended in the following form:

(4) $$\begin{array}{cc} \min_{X}\; & \mathrm{Tr}\left(X^{⊤}\left(S_{w}-\mu S_{b}\right)X\right)\\ s.t.\;\; & X^{⊤}X=I. \end{array}$$

However, LDA still has some shortcomings. For example, LDA can only reduce the dimensionality of data with a category of *k* to k−1 at most. Therefore, LDA cannot be used when reducing the dimensionality below k−1. In addition, if the original sample size is too small, the dimensionality reduction results of LDA are prone to overfitting. Therefore, a common modification is to add sparse constraints to LDA, commonly known as SLDA. In common SLDA methods, the $L_{1}$-norm is applied in LDA to induce sparsity, which can remove redundant features from the data and improve the performance of video image processing. The formulation of SLDA, which introduces the $L_{2,1}$-norm, is expressed as follows:

(5) $$\begin{array}{cc} \; & \min_{X}\;\mathrm{Tr}\left(X^{⊤}\left(S_{w}-\mu S_{b}\right)X\right)+\lambda {∥X∥}_{2,1}\\ \; & \;s.t.\;\;X^{⊤}X=I. \end{array}$$

To effectively eliminate noise and outliers in SLDA and improve robustness in discriminant analysis, reference [14] proposed RSLDA, which is expressed in the form of

(6) $$\begin{array}{cc} \; & \min_{P,X,E}\;\mathrm{Tr}\left(X^{⊤}\left(S_{w}-\mu S_{b}\right)X\right)+\lambda_{1}{∥X∥}_{2,1}+\lambda_{2}{∥E∥}_{1}\\ \; & \;s.t.\;\;R=PX^{⊤}R+E,\;P^{⊤}P=I, \end{array}$$

where ${∥\cdot ∥}_{1}$ is the $L_{1}$-norm. By selecting different parameters of $\lambda_{1}$ and $\lambda_{2}$, RSLDA can select important features and effectively eliminate noise and outliers, thereby achieving excellent performance in the field of image classification.

Another method to improve the performance of SLDA is to incorporate manifold constraints into the optimization problem, such as in the RSMDA method from reference [24], which is represented as

(7) $$\begin{array}{cc} \; & \min_{P,X,E}\;\mathrm{Tr}\left(X^{⊤}\left(S_{w}-\mu S_{b}\right)X\right)+\mathrm{Tr}\left(Q^{⊤}X^{⊤}\left(S_{w}-\mu S_{b}\right)QX\right)+\lambda_{1}{∥X∥}_{2,1}+\lambda_{2}{∥E∥}_{1}\\ \; & \;s.t.\;\;Q=PX^{⊤}Q+E,\;P^{⊤}P=I. \end{array}$$

Inspired by the above methods, this paper proposes an LDA variant that utilizes joint sparsity and manifold constraints. The specific optimization problem will be described in detail in Section 3.

## 3. Methodology

### 3.1. Optimization Problem

In this paper, for the random matrix *X*, the proposed distributed sparse manifold constraints can be expressed as the following problem:

(8) $$\begin{array}{cc} \; & \min_{X}\;\sum_{i=1}^{l}f_{i}\left(X\right)+\lambda g\left(X\right),\\ \; & {s.t.∥X∥}_{2,0}\leq s,X^{T}X=I_{p}, \end{array}$$

where *l* represents the total number of distributed representations of *X*. Distributed sparse manifold constraints can fully utilize the spatial information of the current extended variables, further improving the interpretability of variables from the process monitoring data. Therefore, combined with regular LDA, distributed sparse manifold linear discriminant analysis (DSCLDA) is proposed, which can fully utilize the local and global information of process monitoring observations and take into account both causal and structural relationships between variables.

In this model, $f_{i}\in R^{n\times p}\to R\left(i=1,2,\ldots ,l\right)$ is the given Lipschitz locally continuous function, and $g\in R^{n\times p}\to$ is the given global function. ${∥X∥}_{2,0}\leq s$ is introduced as the sparse constraint, and $X^{T}X=I_{p}$ is used as the manifold constraint. Substituting problem (4) into the distributed sparse constraint yields

(9) $$\begin{array}{cc} \; & \min_{X}\;\sum_{i=1}^{l}f_{i}\left(X\right)+\lambda g\left(X\right),\\ \; & s.t.\;\;X=A+{E,∥X∥}_{2,0}\leq s,X^{T}X=I_{p}. \end{array}$$

### 3.2. Optimization Algorithm

To obtain an effective algorithm, the distributed variable $X_{i}$ and the global variable *Y* are introduced to transform problem (8) into

(10) $$\begin{array}{cc} \; & \min_{X_{i}}\;\sum_{i=1}^{l}f_{i}\left(X_{i}\right)+\lambda g\left(Y\right),\\ \; & s.t.\;\;X_{i}=A_{i}+E_{i},∥X_{i}{∥}_{2,0}\leq s,Y^{T}Y=I_{p}, \end{array}$$

where $X_{i}$ represents the variables of the *i*th distribution. In problem (10), the sparse constraint only includes the local variable $X_{i}$, and the manifold constraint only includes the global variable *Y*. Therefore, further consideration can be given to the optimization problem of the following penalty function:

(11) $$\begin{array}{cc} \; & \min_{X_{i},Y}\;\sum_{i=1}^{l}f_{i}\left(X_{i}\right)+\lambda g\left(Y\right)+\sum_{i=1}^{l}\mu_{i}∥X_{i}-Y{∥}_{F}^{2},\\ \; & s.t.\;\;X_{i}=A_{i}+E_{i},∥X_{i}{∥}_{2,0}\leq s,Y^{T}Y=I_{p}, \end{array}$$

in which $\mu_{i}$ is the penalty parameter corresponding to each branch.

#### 3.2.1. Updating $X_{i}$

Problem (30) is an NP-hard problem, and there is no explicit solution. Inspired by the Newton hard threshold tracking method, the proposed optimization algorithm extends it to matrices. Assuming the objective function is $h_{i}\left(X_{i}\right)$, then the gradient of this function is represented as

(12) $$\begin{array}{c} \nabla h_{i}\left(X_{i}\right)=\nabla f_{i}\left(X_{i}\right)+2\mu_{i}\left(X_{i}-Y\right). \end{array}$$

The Hessian expression of problem (12) can be written as

(13) $$\begin{array}{c} \nabla^{2}h_{i}\left(X_{i}\right)=\nabla f_{i}\left(X_{i}\right)+2\mu_{i}I_{np}. \end{array}$$

If $X_{i}\in P_{ʃ}\left(X_{i}-\alpha_{i}\nabla h_{i}\left(X_{i}\right)\right)$ is satisfied (where $\alpha_{i}>0$ is the step size parameter), then $X_{i}$ can be considered as the stable point for problem (29). Let $T_{S}\left(X_{i},\alpha_{i}\right)$ represent the set of indicators for the first *s* rows of $X_{i}-\alpha_{i}\nabla h_{i}\left(X_{i}\right)$ under $L_{2}$-norm constraints; then, for any $T_{i}\in T_{s}\left(X_{i},\alpha_{i}\right)$, this satisfies a nonlinear relationship, which is written as

(14) $$\begin{array}{c} H_{i}\left(X_{i},T_{i}\right)=\left(\begin{array}{c} {\left(\nabla h_{i}\left(X_{i}\right)\right)}_{T_{i}}\\ {\left(X_{i}\right)}_{{\bar{T}}_{i}} \end{array}\right)=0, \end{array}$$

in which ${\left(\nabla h_{i}\left(X_{i}\right)\right)}_{T_{i}}$ represents the submatrix in $\nabla h_{i}\left(X_{i}\right)$, and $T_{i}$ is the corresponding indicator set. ${\left(X_{i}\right)}_{{\bar{T}}_{i}}\in R^{n-a}\times p$ indicates the submatrix in $X_{i}$ with ${\bar{T}}_{i}$ as the indicator set. The gradient of $H_{i}\left(X_{i},T\right)$ in $X_{i}$ can be expressed as

(15) $$\begin{array}{c} \nabla H_{i}\left(X_{i},T\right)=\left[\begin{array}{cc} {\left(\nabla^{2}h_{i}\left(X_{i}\right)\right)}_{T_{i}T_{i}}\; & {\left(\nabla^{2}h_{i}\left(X_{i}\right)\right)}_{T_{i}{\bar{T}}_{i}}\\ O_{(n-s)p\times sp}\;\;\;\;\; & I_{(n-s)p} \end{array}\right], \end{array}$$

where ${\left(\nabla^{2}h_{i}\left(X_{i}\right)\right)}_{T_{i}T_{i}}\in R^{sp\times sp}$ represents the Hessian submatrix with the indicator set $T_{i}T_{i}$. Define

(16) $$\begin{array}{c} X_{i}\left(\alpha \right)=\left(\begin{array}{c} {\left(X_{i}\right)}_{T_{i}}+\alpha {\left(D\right)}_{T_{i}}\\ {\left(O\right)}_{T_{i}} \end{array}\right), \end{array}$$

where *D* represents the descending direction. The minimum $X_{i}$ can be obtained using a sparse proximal gradient (SpaPG). The descending direction *D* is obtained from

(17) $$\begin{array}{c} \nabla H_{i}\left(X_{i},T\right)\mathrm{vec}\left(D\right)=-\mathrm{vec}\left(H_{i}\left(X_{i},T\right)\right), \end{array}$$

Then, the k+1th $X_{i}$, $X_{i}^{k+1}$, should be represented as

(18) $$\begin{array}{c} X_{i}^{k+1}=X_{i}^{k}\left(\alpha_{i}^{k}\right), \end{array}$$

in which $\alpha_{i}^{k}=\rho^{\tau }$, while τ is the smallest positive integer that satisfies the following equation, written as

(19) $$\begin{array}{c} h_{i}\left(X_{i}\left(\rho^{\tau }\right)\right)\leq h_{i}\left(X_{i}\right)+\sigma \rho^{\tau }\langle \nabla h_{i}\left(X_{i}\right),D^{k}\rangle \end{array}$$

#### 3.2.2. Updating *Y*

Set $M=\{T|Y^{T}Y=I_{p}\}$; then, the tangent space of manifold M at *Y* is expressed as $T_{Y}M=\left\{Z|Z^{T}Y+Y^{T}Z=0\right\}$. Assuming the objective function is ϕ(Y), it has the following approximation function:

(20) $$\begin{array}{c} \phi \left(Y^{k}\right)+\langle \nabla \phi \left(Y^{k}\right),Y-Y^{k}\rangle +\frac{1}{2t}∥Y-Y^{k}{∥}_{F}^{2}, \end{array}$$

where $\frac{1}{L}\geq t>0$ is a parameter. To obtain the descending direction *D*, define

(21) $$\begin{array}{cc} \min_{D\in R^{R\times P}}\; & \langle \nabla \phi \left(Y^{k}\right),D\rangle +\frac{1}{2t}{∥D∥}_{F}^{2}\\ s.t.\;\; & D\in T_{Y^{k}}M, \end{array}$$

Based on the definition of $T_{Y^{k}}M$, set $D^{T}Y^{k}+Y^{kT}D=0$, and Equation (21) can be represented as

(22) $$\begin{array}{cc} \min_{D\in R^{R\times P}}\; & \langle \nabla \phi \left(Y^{k}\right),D\rangle +\frac{1}{2t}{∥D∥}_{F}^{2}\\ s.t.\;\; & D^{T}Y^{k}+Y^{kT}D=0, \end{array}$$

Based on Equation (22), the Lagrange function can be obtained, which is written as

(23) $$\begin{array}{cc} L(D,\Lambda ) & =\langle \nabla \phi \left(Y^{k}\right),D\rangle +\frac{1}{2t}{∥D∥}_{F}^{2}\\ \; & -\langle \Lambda ,D^{T}Y^{k}+Y^{kT}D\rangle , \end{array}$$

in which $\Lambda \in R^{n\times p}$ is the Lagrange multiplier. Then, the corresponding Karush–Kuhn–Tucker (KKT) system for the Lagrangian function above is represented as

(24) $$\begin{array}{c} \begin{array}{c} \left\{\begin{array}{c} 0\in \partial_{D}L\left(D,\Lambda \right),\\ 0=D^{T}Y^{k}+Y^{kT}D \end{array}\right. \end{array} \end{array}$$

By synthesizing Equation (24), the optimization problem for {D,Λ,Y} can be obtained, which is written as

(25) $$\begin{array}{c} D{\left(\Lambda \right)}^{T}Y^{k}+Y^{kT}D\left(\Lambda \right)=0. \end{array}$$

Equation (25) can be solved using the manifold proximal gradient (ManPG) algorithm, and the k+1th *Y* can be represented as

(26) $$\begin{array}{c} Y^{k+1}=R_{Y^{k}}\left(\gamma^{k}D^{k}\right), \end{array}$$

in which the mapping $R_{Y}:T_{Y}M\to M$ represents Retraction. $R_{Y}$ maps the vectors in the tangent space to the manifold, allowing the problem to maintain orthogonality during the optimization. In Equation (26), $\gamma^{k}=\gamma \eta^{q}$, and *q* is the smallest positive integer that satisfies the following equation, expressed as

(27) $$\begin{array}{c} \phi \left(Y^{k+1}\right)\leq \phi \left(Y^{k}\right)-\frac{\gamma \eta^{q}}{2t}∥D^{k}{∥}_{F}^{2}. \end{array}$$

### 3.3. Convergence Analysis

According to the updates of $X_{i}$ and *Y*, the optimization algorithm of Equation (11) can be expressed as Algorithm 1. In addition, according to the literature [36], if $\left(X_{i}^{*},Y^{*}\right)$ satisfies

(28) $$\begin{array}{c} \left\{\begin{array}{c} 0\in P_{S}\left(\nabla f_{i}\left(X_{i}^{*}\right)+\mu_{i}\left(X_{i}^{*}-Y^{*}\right)\right),\\ 0\in P_{M}\left(\lambda \nabla g\left(Y^{*}\right)-\mu_{i}\left(X^{*}-Y^{*}\right)\right), \end{array}\right. \end{array}$$

then $\left(X_{i}^{*},Y^{*}\right)$ can be considered as the stable point of Equation (11). The experimental verification of the convergence analysis can be found in Section 4.5.

### 3.4. Complexity Analysis

To verify how distributed sparse constraints can enhance the performance of existing methods, this section compares the complexity and computational cost of the proposed method with that of the baseline LDA method. For Equation (9), given the original data with a dimensionality of *d* and *n* samples, the computational complexity of the objective function is $O(nd^{2})$. The sparse constraint ${∥X∥}_{2,0}\leq s$, which checks the number of non-zero elements, has a complexity of O(nd). The manifold constraint $X^{T}X=I_{p}$ implies that *X* is an orthogonal matrix, which, enforcing orthogonality through methods such as QR decomposition, has a complexity of $O(nd^{2})$. Therefore, the overall complexity of the proposed method is $O(nd^{2})$. In contrast, the computational complexity of traditional LDA-based methods is primarily determined by calculating the within-class scatter matrix $S_{w}$, the between-class scatter matrix $S_{b}$, and solving the generalized eigenvalue problem. The complexity of computing $S_{w}$ is $O(nd^{2})$, while that of computing $S_{b}$ is $O(d^{2})$, due to the calculation based on class means and the global mean. The complexity of solving the eigenvalues and eigenvectors of $S_{w}^{-1}S_{b}$ is $O(d^{3})$, which is the most time-consuming part of LDA. The overall complexity of LDA is $O(nd^{2}+d^{3})$. The proposed distributed sparse constraint method demonstrates superior computational efficiency over traditional LDA methods by reducing the overall complexity from $O(nd^{2}+d^{3})$ to $O(nd^{2})$ through the enforcement of sparsity and orthogonality constraints, thereby eliminating the most time-consuming generalized eigenvalue problem in LDA.
**Algorithm 1** Optimization algorithm for (11)**Input:** Data *X*, parameters *s*,*l*,λ,$\mu_{i}>0$.**Initialize:** Data $Y^{0}$, parameter k=0.**Output:** Data *Y*.**While** not converged **do**1:According to Algorithm 2, update $X_{i}^{k+1}$ by(29)$$\begin{array}{c} \min_{X_{i}}f_{i}\left(X_{i}\right)+\mu_{i}∥X_{i}-Y{∥}_{F}^{2},\;s.t.\;X_{i}=A_{i}+E_{i},∥X_{i}{∥}_{2,0}\leq s. \end{array}$$2:According to Algorithm 3, update $Y^{k+1}$ by(30)$$\begin{array}{c} \min_{Y}\;\lambda g\left(Y\right)+\sum_{i=1}^{l}\mu_{i}∥X_{i}-Y{∥}_{F}^{2},\;s.t.\;Y^{T}Y=I_{p}. \end{array}$$3:If the process meets the shutdown criteria $∥X_{i}{∥}_{2,0}\leq s$, stop; Otherwise, let k=k+1 and return to Step 1.**End while**

**Algorithm 2** Optimization algorithm for (12)
**Input:** Data *X*, parameters μ,α>0, ρ∈(0,1), σ∈(0,1/2).
**Initialize:** $X_{i}^{0}$, $T_{i}^{0}\in T_{S}\left(X_{i}^{0},\alpha \right)$, when k=0.

**Output:** $X_{i}^{k}$


**While** not converged **do**
1:Obtain the nonlinear relationship $H_{i}\left(X_{i}^{k},T^{k}\right)$ and the gradient $\nabla H_{i}\left(X_{i}^{k},T_{i}^{k}\right)$, according to (14), (15);2:Obtain the descent direction $D^{k}$, according to (17);3:Update the local variable $X_{i}^{k+1}$, according to (9);4:If the process meets the shutdown criteria, stop; Otherwise, let k=k+1, update $T_{i}^{k}\in T_{S}\left(X_{i}^{k},\alpha^{k}\right)$, and return to Step 1.

**End while**



**Algorithm 3** Optimization algorithm for (20)
**Input:** Parameters γ,t>0,η∈(0,1).
**Initialize:** $Y^{0},\Lambda^{0}$, k=0.
**Output:** $X^{k},Y^{k}$.
**While** not converged **do**
1:Obtain the descent direction *D*, according to (25);2:Update the global variable $Y^{k+1}$, according to (26),3:If the process meets the shutdown criteria, stop; Otherwise, let k=k+1 and return to Step 1.

**End while**



## 4. Simulation Studies

**Figure 1:**
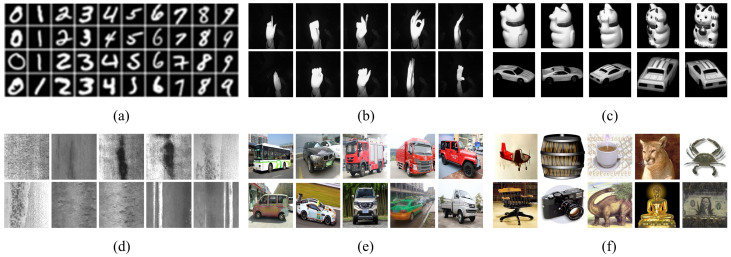
Some image examples from the datasets used in the experiment. (**a**) Mnist, (**b**) Hand Gesture Recognition, (**c**) COIL20, (**d**) NEU surface defects, (**e**) Car_image, (**f**) Caltech-101.

In the experiments, DSCLDA was compared with traditional LDA and six LDA variants, including AFLDA [17], ERSLDA [37], RSLDA+IIKC [15], RSMDA [24], RSLDA [14], SULDA [13], and SLDA [12]. The optimization problem and constraint of each method are shown in Table 1. The datasets used in the experiments in this paper are shown in Table 2, and examples of each dataset are shown in Figure 1. In this experiment, a self-built vehicle dataset, called the CAR_image dataset, was introduced.

### 4.1. Experiment Settings

Due to the fact that the datasets were divided into *D* parts as data nodes for distributed computing during the experiment, D was added before all method names to indicate distributed performance, such as DERSLDA and DRSLDA. In the simulation verification, each method was executed 10 times, with different random samples selected from the same dataset for each run; then, the average classification accuracy was calculated. To improve computational efficiency, all datasets were preconverted into grayscale images. In addition, to improve computational efficiency and achieve better classification accuracy, this experiment used PCA to perform dimensionality reduction on all image datasets, retaining 95% of the original data information. Furthermore, due to the large and inconsistent image sizes in the Car_image dataset, the unified resolution of the images in this dataset was 64×128.

**Figure 2:**
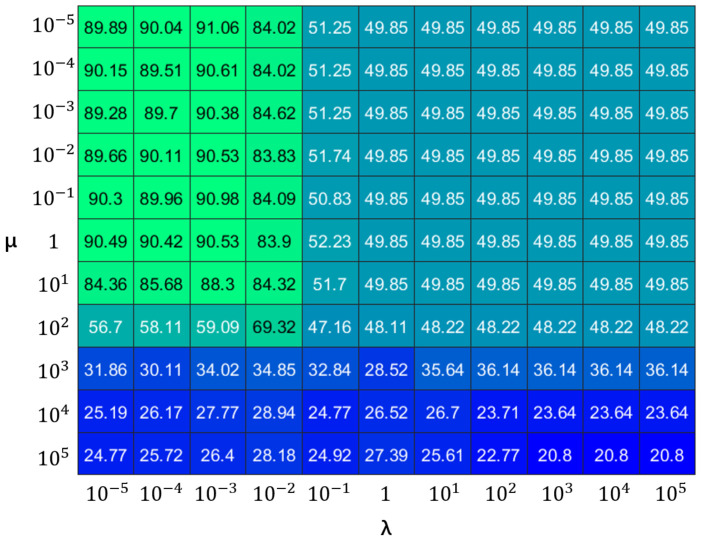
Parameter cross-validation on the COIL20 image dataset. The parameters λ and μ are derived from Equation (11) In this figure, green indicates high value and blue indicates low value.

In selecting experimental parameters, the selection of the parameters λ and μ was carried out through a ten-fold cross-validation method based on the content and size of each dataset. The range of the parameters λ and μ is denoted as $10^{-5},10^{-4},10^{-3},10^{-2},10^{-1},1,10^{1},$$10^{2},10^{3},10^{4}$, and $10^{5}$. Prior to numerical validation, a strategy of fixing the value of λ while varying μ was employed to ascertain the corresponding accuracy for each configuration, serving as a basis for evaluation. The experimental results on the COIL20 image dataset are shown in Figure 2. Based on the experimental results, it can be determined that the selection of the parameters λ and μ should be within the range of $10^{-5}$, $10^{-4}$, $10^{-3}$, and $10^{-2}$ to achieve better image processing performance. Specifically, for the COIL20 image dataset, the most suitable parameter combination was identified as $10^{-3}$ and $10^{-5}$, and a similar method for parameter selection was applied to other datasets under investigation. In addition, the shutdown criterion in this experiment was set so that 100 iterations would be reached or the overall objective function value would be less than $10^{-3}$.

### 4.2. Experiment Based on Sample Size

This experiment used the k nearest neighbors (KNN) classifier to analyze the classification accuracy of the dimensionality reduction results of various methods. The knn classifier is a supervised machine learning algorithm that assigns a new data point to the class most common among the *k* nearest neighbors in the feature space, based on a distance metric such as the Euclidean distance. In this experiment, four different sample sizes were randomly selected for each dataset as the training set, and the remaining samples were used as the testing set. The classification experiment results under different sample sizes are shown in Table 3, where the highest-performing results are highlighted in bold. The simple image datasets used in the experiment, including the Mnist dataset, Hand Gesture Recognition dataset, and COIL20 dataset, have simple content, a monotonous background, and obvious features. Therefore, each method could achieve better classification performance on the above three datasets. The image features of the NEU surface defect dataset, Car_image dataset, and Caltech-101 dataset are relatively complex or have a high proportion of the background, resulting in relatively low classification accuracy for each model on these datasets. However, the experimental results show that the DSCLDA model still had improvements in the classification performance compared to other methods on these datasets.

Compared with other methods, DSCLDA improved by at least 0.51% on the Mnist dataset; improved by at least 0.44% on the Hand Gesture Recognition dataset; improved by at least 0.85% on the COIL20 image dataset; improved by at least 0.86% on the NEU surface defect dataset; improved by at least 2.16% on the Car_image dataset; and improved by at least 0.55% on the Caltech-101 image dataset. The classification performance of DSCLDA was further improved on two difficult datasets, namely the NEU surface defect dataset and the Car_image dataset. The results can be explained by the fact that the DSCLDA model, which simultaneously extracts features from both global and local structures, can obtain more representative feature data when processing complex images or images with unclear features, thereby achieving better classification performance. The experimental results also demonstrate that DSCLDA divides process monitoring data into multiple data nodes and performs distributed parallel operations, which not only improves computational efficiency but also provides better adaptation to the processing needs of large-scale data.

Compared to other methods, the average classification performance of the proposed DSCLDA method improved by at least 0.90%, which proves that the proposed method achieves satisfactory classification performance by introducing joint sparse and manifold constraints. In addition, compared with DRSLDA, DRSMDA, DRSLDA+IIKC, and DERSLDA, the proposed DSCLDA still had a significant improvement, indicating that the proposed method can demonstrate advantages when compared with some of the latest SLDA variants.

### 4.3. Experiment Based on the Number of Dimensions

**Figure 3:**
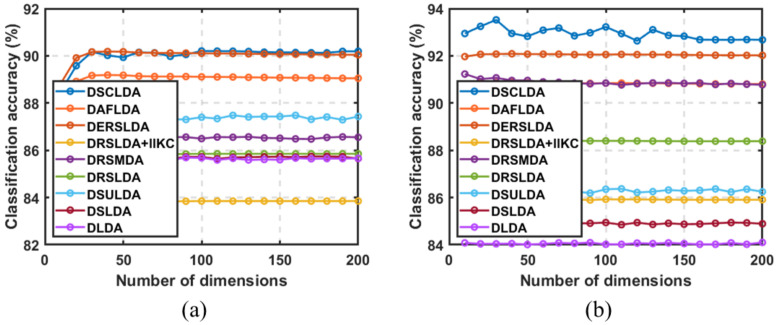
Classification accuracy on the Mnist dataset. (**a**) Number of samples: 50; (**b**) number of samples: 100.

**Figure 4:**
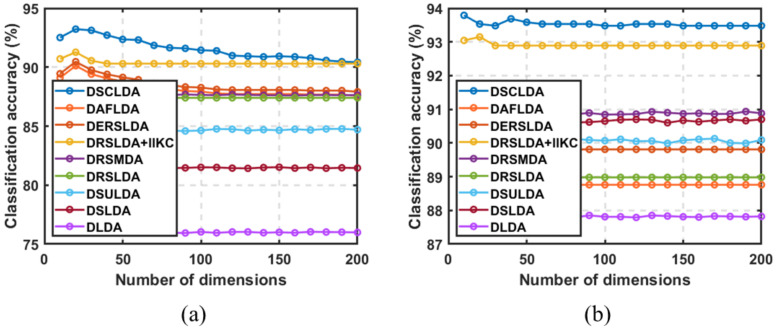
Classification accuracy on the Hand Gesture Recognition dataset. (**a**) Number of samples: 4; (**b**) number of samples: 6.

**Figure 5:**
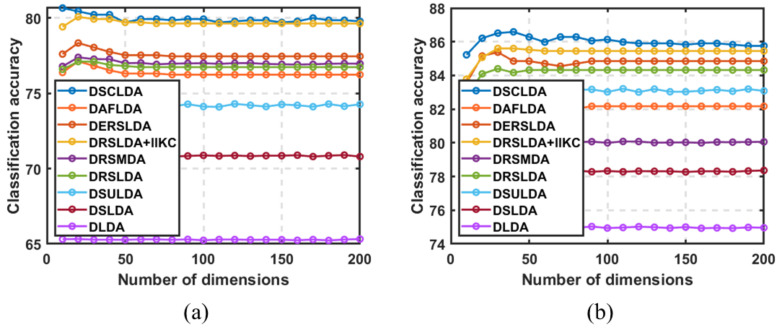
Classification accuracy on COIL20 image dataset. (**a**) Number of samples: 4; (**b**) number of samples: 6.

**Figure 6:**
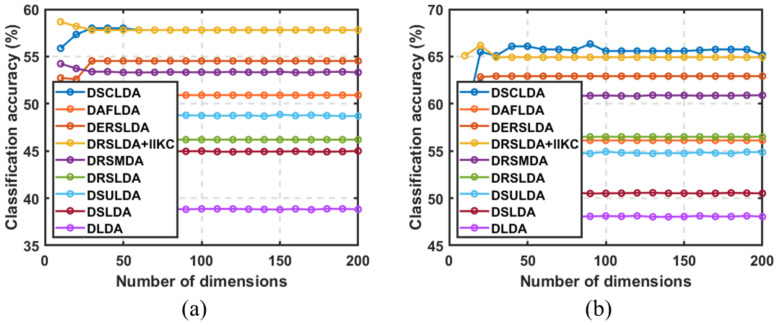
Classification accuracy on the NEU surface defect dataset. (**a**) Number of samples: 50; (**b**) number of samples: 100.

**Figure 7:**
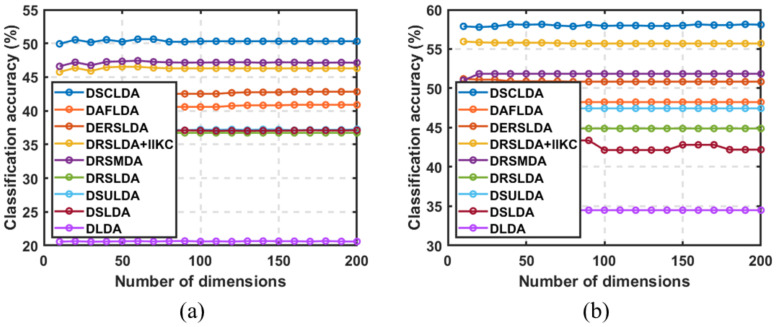
Classification accuracy on the Car_image dataset. (**a**) Number of samples: 10; (**b**) number of samples: 20.

**Figure 8:**
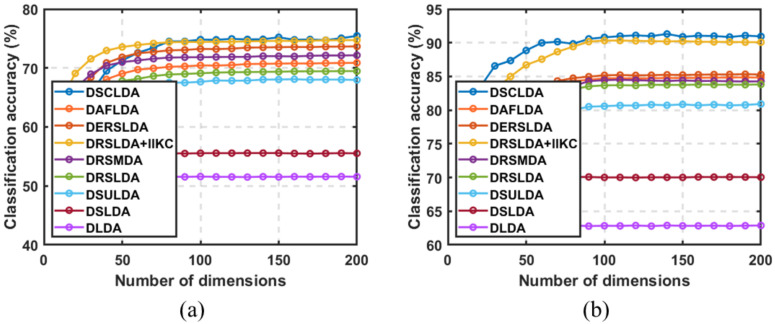
Classification accuracy on the Caltech-101 image dataset. (**a**) Number of samples: 10; (**b**) number of samples: 20.

In this experiment, (50,100), (4,6), (4,6), (50,100), (10,20), and (10,20) samples were selected as training sets for each type on the six public image datasets, and the remaining samples were used as testing sets with dimensions ranging from 5 to 200. The classification experiment results are shown in Figure 3, Figure 4, Figure 5, Figure 6, Figure 7 and Figure 8. The experimental results indicate that the proposed DSCLDA method achieved relatively better classification performance on the six publicly available datasets mentioned above. From the experimental results, it can be seen that the classification performance of DLDA and DSLDA is very sensitive to the choice of dimensionality. As the dimensionality increases, the classification performance of these two methods may even even decrease. However, the proposed DSCLDA method can still maintain classification accuracy in the presence of dimensional changes. The experimental results demonstrate the flexibility of the proposed method in dimension selection. For the NEU surface defect and Caltech-101 image datasets, the classification performance of DSCLDA did not show significant improvement compared to that of other methods because the features of these two datasets are relatively complex and not clear enough, resulting in similar classification results for the above methods. However, on several other publicly available datasets, the proposed DSCLDA method still performed relatively better in terms of its classification accuracy. DSCLDA normalizes local features by constructing $L_{2,0}$-norm sparse constraints, preserves the local structure of variables, and utilizes manifold constraints to capture causal correlations between global variables, reducing data structure loss during the projection process.

### 4.4. Experiments with Deep Learning Methods

Deep learning methods, such as Transformer-based feature extraction models, provide new perspectives and powerful tools for feature extraction and dimensionality reduction, which can provide valuable benchmarks. These deep learning methods typically have better feature-learning capabilities and stronger robustness and can achieve excellent performance on large-scale datasets. Therefore, this paper also compares DSCLDA with deep learning-based dimensionality reduction techniques, such as R3D-CNN [43], I3D [44], and Transformer [45], to demonstrate its broader applicability in different scenarios. Through experiments on the Hand Gesture Recognition (HGR) and CIFAR-100 [46] datasets, we validated the advantages of DSCLDA in feature extraction and dimensionality reduction, as well as its competitiveness with deep learning methods. Table 4 demonstrates that the gesture recognition dataset may have certain limitations, such as its number of samples, diversity, and representativeness. On smaller datasets, simpler or more traditional models, such as DSCLDA, may perform better because of their lower complexity. On the other hand, models supported by deep learning methods may be more suitable for handling large and complex datasets, capable of capturing more subtle patterns and relationships.

### 4.5. Convergence Analysis

**Figure 9:**
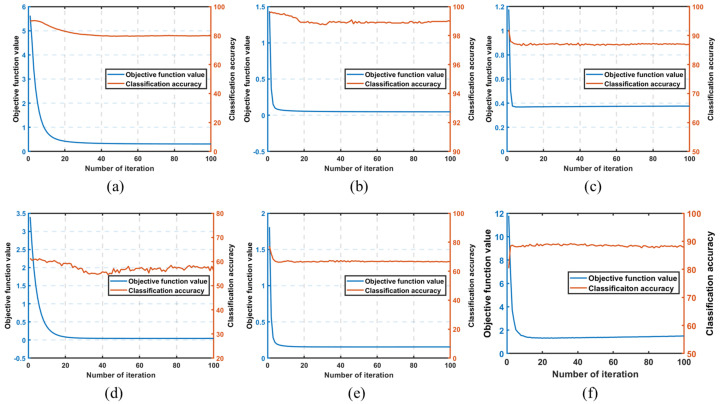
The relationship between the objective function value, classification accuracy, and the number of iterations. (**a**) Mnist, (**b**) Hand Gesture Recognition, (**c**) COIL20, (**d**) NEU surface defects, (**e**) Car_image, (**f**) Caltech-101.

This section describes how we conducted experimental verification of the convergence analysis proposed in Section 3.3. In the proposed DSCLDA method, the most computationally expensive step is the calculation of the projection matrix *X*, while the most computationally intensive task is the process of solving the inverse matrix, which significantly affects the computational efficiency of DSCLDA. In this experiment, the computational efficiency of DSCLDA was reflected in the speed of function value reduction and the convergence speed of the classification accuracy. In order to visually demonstrate the convergence of the proposed DSCLDA method, Figure 9 shows the curves of the objective function value and classification accuracy of the functions. As the number of iterations increased, the objective function value of the proposed DSCLDA method rapidly decreased and reached its minimum value, and the classification accuracy also reached its maximum value and converged within 30 iterations. The experimental results validate the fast convergence of DSCLDA.

### 4.6. t-SNE Comparison

**Figure 10:**
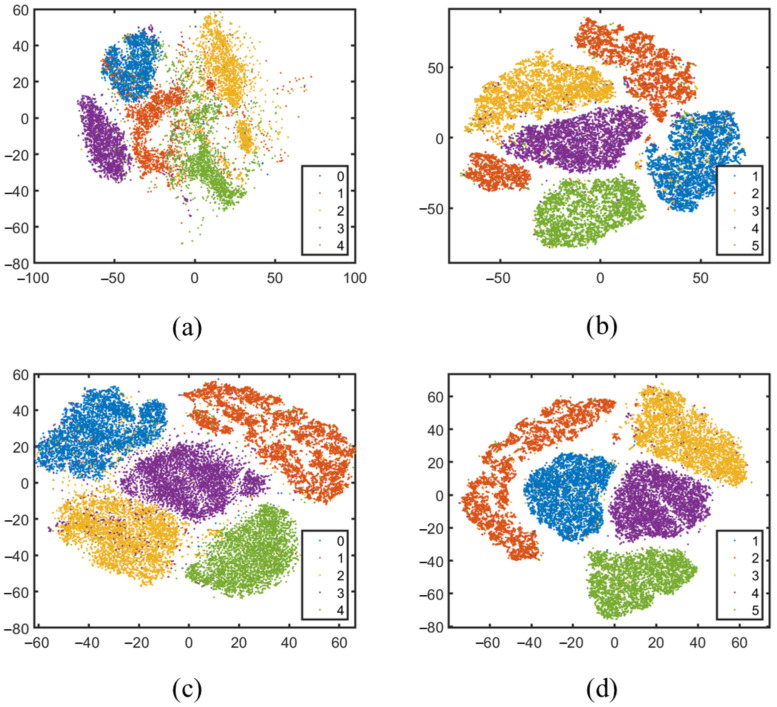
The data distribution displayed using the t-SNE method. The images correspond to (**a**) the local data of the original Mnist dataset; (**b**) the distribution of the corresponding data after DRSLDA projection; (**c**) the distribution of the corresponding data after DERSLDA projection; (**d**) and the corresponding data distribution projected through DSCLDA.

In addition, to further validate the principle and effectiveness of the proposed method, the t-SNE method was utilized to visualize the data distribution before and after projection. The experiment used the top five types of data from the Mnist dataset and randomly selected 100 samples for each type as the training set and the remaining samples as the testing set. The corresponding classification accuracies of each method were 85.85% (DRSLDA), 90.10% (DERSLDA), and 90.20% (DSCLDA), respectively. The experimental results are shown in Figure 10. It can be seen that when not projected, the inter-class and intra-class distributions of the Mnist dataset were not significant. When using the DRSLDA method for projection, it can reduce the distance between types and increase the distance between different types, but DRSLDA cannot fully classify all data, and this classification method is not satisfactory in terms of distribution. With the introduction of sparse constraints, the inter-class distance between different types of data becomes larger, while the intra-class spacing becomes smaller. In the t-SNE distribution of the proposed DERSLDA method, the intra-class spacing is relatively small, but the inter-class distance is not large enough, so there is still a possibility of data confusion during the classification process. In the t-SNE distribution of the proposed DSCLDA method, the distance between different types is the largest, and the distance between types is the smallest, such as between type 1 and type 2, which is more conducive to determining the data category during the classification process. The experimental results show that the proposed method has relatively better classification performance.

## 5. Conclusions

In this paper, we constructed a novel distributed sparse manifold constraint and a novel LDA variant, called DSCLDA. The proposed method trains discriminative projections by introducing manifold constraints and $L_{2,0}$-norm sparse constraints, which can obtain the most discriminative features for process monitoring. In addition, in this paper, we designed and developed a novel manifold proximal gradient algorithm to handle the proposed optimization model, while distributed parallel computing could significantly improve computational efficiency. The advantages of DSCs and DSCLDA have been demonstrated through numerical experiments on several public datasets. Compared with other existing LDA methods, the proposed DSCLDA method improves the image classification accuracy by at least 0.90% and also has significant advantages in convergence and feature distribution.

However, the proposed method currently exhibits limitations in terms of its image processing efficiency and feature classification accuracy, necessitating integration with deep learning techniques for improvement. In the future, we will attempt to combine the proposed method with deep learning methods to improve the efficiency of image processing and the accuracy of feature classification. Furthermore, deployment on hardware platforms may be constrained by computational complexity and insufficient flexibility, highlighting the need for further optimization to enhance the processing efficiency and applicability. In addition, this method will also be considered for deployment on hardware to improve the efficiency of the method’s processing and the flexibility of the method’s usage. 

## Figures and Tables

**Table 1:** Information on all comparison methods used in this experiment. The bold method is the proposed method.

Method	Optimization Problem	Constraint
LDA	$\min_{X}\;\mathrm{tr}\left(X^{⊤}\left(S_{w}-\mu S_{b}\right)X\right)$	$s.t.\;X^{⊤}X=I$
SLDA	$\min_{X}\;\mathrm{Tr}\left(X⊤(S_{w}-\mu S_{b})X\right)+\lambda {∥X∥}_{2,1}$	s.t.X⊤X=I
SULDA	minG	$s.t.\;U_{1}^{T}G=\sum_{t}^{-1}P_{1}Z,Z^{T}Z=I$
RSLDA	$\min_{P,X,E}\;\mathrm{Tr}\left(X^{⊤}\left(S_{w}-\mu S_{b}\right)X\right)$	$s.t.\;R=PX^{⊤}R+E,\;P^{⊤}P=I$
	$+\lambda_{1}{∥X∥}_{2,1}+\lambda_{2}{∥E∥}_{1}$	
RSMDA	$\min_{P,X,E}\;\mathrm{Tr}\left(X^{⊤}\left(S_{w}-\mu S_{b}\right)X\right)$	$s.t.\;R=PX^{⊤}R+E,\;P^{⊤}P=I$
	$+\mathrm{Tr}\left(X^{⊤}R^{⊤}\left(L_{w}-L_{b}\right)RX\right)$	
	$+\lambda_{1}{∥X∥}_{2,1}+\lambda_{2}{∥E∥}_{1}$	
RSLDA+IIKC	$\min_{P,X,E}\;\mathrm{Tr}\left(X^{⊤}\left(S_{w}-\mu S_{b}\right)X\right)$	$s.t.\;R=PX^{⊤}R+E,\;P^{⊤}P=I$
	$+\lambda_{1}{∥X∥}_{2,1}+\lambda_{2}{∥E∥}_{1}$	
	$+\alpha \mathrm{Tr}\left(X^{⊤}\left(S_{w}^{\prime }-\mu S_{b}^{\prime }\right)X\right)$	
ERSLDA	$\min_{P,X,E,N}\;\mathrm{Tr}\left(X^{⊤}\left(S_{w}-\mu S_{b}\right)X\right)$	$s.t.\;R=PX^{⊤}R+E+N,\;P^{⊤}P=I$
	$+\lambda_{1}{∥X∥}_{2,p}^{p}+\lambda_{2}{∥E∥}_{p}^{p}+\eta {∥N∥}_{F}^{2}$	
**DSCLDA**	$\min_{X}\;\sum_{i=1}^{d}\mathrm{tr}\left(X^{⊤}\left(S_{i}^{w}-\tau S_{i}^{b}\right)X\right)$	${s.t.\;∥X∥}_{2,0}\leq s,\;X^{⊤}X=I_{p}$

**Table 2:** Information related to the dataset used in this experiment.

Dataset	Image Types	Images	Color Type	Original Resolution
Mnist [38]	10	60,000	Gray	28×28
Hand Gesture Recognition [39]	10	20,000	Gray	240×640
Coil20 [40]	20	1440	Gray	128×128
NEU surface defects [41]	6	1200	Gray	32×32
Car_image	10	200	RGB	800×600 to 5000×3000
Caltech-101 [42]	101	9146	RGB and gray	About 300×200

**Table 3:** The classification accuracy obtained on six datasets. The bold value represents the highest value of the column.

Methods	Mnist				Hand Gesture Recognition				COIL20			
	10	50	100	200	4	5	6	7	3	6	9	12
DLDA	75.20	85.59	84.02	83.78	75.95	80.38	87.81	89.35	65.29	74.97	81.58	80.40
DSLDA	80.42	85.64	84.85	84.46	81.50	83.69	90.69	92.23	70.84	78.28	84.46	84.02
DSULDA	**87.78**	87.34	86.37	93.38	84.76	88.62	90.11	91.65	74.10	83.21	83.88	86.37
DRSLDA	84.03	85.85	88.40	96.58	87.40	89.73	88.98	90.52	76.74	84.32	82.75	86.75
DRSMDA	83.62	86.56	90.77	97.51	87.62	85.49	90.85	92.39	76.96	80.08	84.62	87.30
DRSLDA+IIKC	73.27	83.85	85.92	96.94	90.30	90.86	92.89	94.43	79.64	85.45	86.66	86.94
DERSLDA	86.77	90.10	92.06	97.62	88.12	90.26	89.81	91.35	77.46	84.85	83.58	88.73
DAFLDA	85.91	89.11	90.84	96.34	87.93	88.28	88.76	90.67	76.24	82.17	83.36	85.23
**DSCLDA**	86.92	**90.20**	**92.94**	**97.82**	**90.37**	**91.39**	**93.48**	**95.02**	**79.71**	**85.98**	**87.25**	**90.95**
**Methods**	**NEU Surface Defects**				**Car_IMAGE**				**Caltech-101**			
	**25**	**50**	**75**	**100**	**10**	**15**	**20**	**25**	**10**	**15**	**20**	**25**
DLDA	41.27	38.87	43.26	48.08	20.64	25.00	34.50	44.77	51.54	58.16	62.82	65.21
DSLDA	43.03	44.93	48.15	50.53	37.03	39.93	42.15	42.08	55.56	67.60	70.02	74.32
DSULDA	42.18	48.73	52.45	54.82	37.18	42.73	47.45	48.82	67.89	77.09	80.69	86.11
DRSLDA	42.73	46.20	50.89	56.50	36.73	40.20	44.89	50.50	69.22	83.60	83.70	87.04
DRSMDA	52.18	53.33	57.85	60.83	47.18	47.33	51.85	54.83	71.86	83.33	84.51	86.25
DRSLDA+IIKC	52.30	**57.80**	61.70	64.92	46.30	51.80	55.70	59.92	74.45	87.52	90.32	91.02
DERSLDA	47.52	54.53	55.85	62.92	42.52	49.53	50.85	56.92	73.20	85.10	85.20	88.25
DAFLDA	45.64	50.91	52.68	56.12	40.58	46.37	48.25	53.76	70.45	83.68	84.71	85.99
**DSCLDA**	**54.55**	**57.80**	**62.22**	**65.58**	**50.32**	**53.57**	**57.99**	**61.35**	**74.79**	**88.28**	**90.98**	**91.47**

**Table 4:** The accuracy of DSCLDA, R3D-CNN, I3D, and Transformer on the HGR and CIFAR-100 datasets. The bold value represents the highest value of the column.

HGR		CIFAR-100	
Method	Acc. (%)	Method	Acc. (%)
DSCLDA	**90.37**	DSCLDA	63.45
R3D-CNN	83.80	R3D-CNN	90.62
I3D	85.70	I3D	94.82
Transformer	87.60	Transformer	95.03

## Data Availability

All datasets used are available online with open access.
